# Considerations for sociocultural adaptations of a mindfulness-based program within a low socioeconomic setting in Cape Town, South Africa

**DOI:** 10.1186/s12906-025-05122-3

**Published:** 2025-12-30

**Authors:** Sarah Foale, Soraya Seedat, Tanya Heyns

**Affiliations:** 1 Department of Psychiatry, Faculty of Medicine and Health Sciences, University of Stellenbosch, Stellenbosch, South Africa; 2 Department of Nursing Science, Faculty of Health Sciences, University of Pretoria, Pretoria, South Africa

**Keywords:** Adaptation, Low resource, Low socioeconomic status, Mindfulness, Mindfulness-based

## Abstract

**Background:**

The effectiveness of mindfulness-based programs in cultivating a sense of well-being has been demonstrated in many populations globally. However, mindfulness research is lacking in low socioeconomic contexts. This study explored how a mindfulness-based program may be adapted to be feasible, acceptable, and accessible to participants in a low socioeconomic setting in Cape Town, South Africa.

**Methods:**

Using purposive and snowball sampling and semi-structured interviews, the perspectives of 14 stakeholders in the field of mindfulness were explored. The data were thematically analyzed.

**Findings:**

Stakeholders agreed that MBPs could be adapted to increase acceptability, accessibility, and feasibility for participants. The themes that emerged included strategies to encourage attendance and retention, program structure, program content, trauma sensitivity, the qualities and training of the facilitator, communication strategies, the language used, and the approach to translation.

**Conclusion:**

Stakeholders agreed that adapted mindfulness-based programs may be beneficial to participants in low socioeconomic contexts if appropriately and sensitively adapted to the needs of the population and context. Further research is invited into the nuances of what, where, when, and how mindfulness-based programs are offered in low-resource and especially high-risk trauma environments, such as those in South Africa.

**Supplementary Information:**

The online version contains supplementary material available at 10.1186/s12906-025-05122-3.

## Introduction

South Africa is characterized by the world’s greatest disparity between rich and poor individuals [1], between those living in well-resourced circumstances and those whose lives are poorly resourced in almost every respect, from employment to housing to education to healthcare [2]. The field of mindfulness is no exception to this disparity. While the first author’s experience of working in the field suggests that mindfulness-based programs (MBPs) are offered in low-resource contexts, there is a paucity of research on MBPs in low socioeconomic settings in South Africa and internationally [3]. This study arose from a deep concern about the inequity of access to mindfulness as an evidence-based modality to enhance well-being.

Mindfulness is the clear, undistracted and nonjudgmental attention to consciousness [4]. The practice of mindfulness involves engaging with our thoughts, feelings and bodily sensations as they arise, with an attitude of nonjudgment, acceptance, curiosity and compassion directed towards alleviating distress and suffering [5–12]. Mindfulness may be cultivated by establishing regular formal mindfulness practices such as sitting practice or yoga, and informal practices such as eating or washing hands. The regular practice of mindfulness develops the innate and universal human capacity for bringing attention to the lived experience of the present moment [8], which in turn may improve our sense of well-being and that of our communities and the world at large [13].

MBPs offer training to develop mindfulness and integrate regular practice into daily life to increase participants’ capacity for making choices that alleviate suffering and result in a deepened sense of well-being [7, 8, 10]. For a program to be considered mindfulness-based, it should be structured around the “nonnegotiable” foundational principles of mindfulness, such as the type of practices, the focus of the practices, the enquiry process, and the attitudes of mindfulness [8]. However, MBPs may also be modified or adapted according to the “negotiable” elements of a program, such as the length of the practices or the duration of the program [7].

The foundational structures and practices of mindfulness are rooted within Asia’s wisdom traditions and are an integral part of Buddhist culture [14]. Contemporary Western practices, however, generally adopt a secular stance [11, 12]. This secular approach ensures that the program and practices are grounded within contemporary scientific approaches to managing physical and mental health and supporting general well-being, particularly within neuroscience and neuropsychology [7].

The contemporary mindfulness movement began – at least in part – as an outcome of the work and personal meditation experience of microbiologist Dr. Jon Kabat-Zinn. In 1979, at the University of Massachusetts Medical School, Kabat-Zinn developed the Mindfulness-based Stress Reduction program (MBSR): a manualized, experiential 8-week training program that focuses on using a specific range of formal practices (bodyscan, sitting practice, open awareness and various forms of mindful movement) and informal practices (such as washing hands, eating, etc.) to cultivate mindful awareness in everyday life [10, 15]. The initial purpose of the program was to offer a compassionate and empowering approach to patients dealing with otherwise unsolvable pain, chronic illness and stress-related health conditions, as well as psychological and emotional stress and anxiety [10, 11].

In the following years, research on the mechanisms and benefits of mindfulness practices for physical and psychological health and well-being has increased markedly [13]. Numerous MBPs have been developed that offer mindfulness training within various settings and for multiple purposes, including anxiety, depression, eating disorders, relapse prevention, business, leadership, trauma, education, childbirth and parenting [16–19]. However, much of this research has been conducted among what may be considered WEIRD populations: Western, educated, industrialized, rich, and Democratic, and particularly white, female, and middle- to upper-class populations [15, 20–23]. Many MBPs require significant financial commitment from participants and access to other resources, such as convenient and affordable transport, access to the internet, and time. Consequently, mindfulness training appears to be reserved for socioeconomically robust populations.

Although the first author’s experience in the field suggests that MBPs are offered within low-resource communities, there is a lack of documented research, as highlighted by Foale et al. [3]. There is no clear protocol regarding what works for whom, where, and how, concerning MBPs within low socioeconomic settings, whether internationally or in South Africa. There is thus a need to explore how the negotiable elements of MBPs may be adapted to benefit participants’ experiences of well-being within a low socioeconomic setting. The findings may contribute to developing a body of knowledge on MBP adaptation in low-resource settings, both locally and worldwide. In the long term, appropriately adapted MBPs may enable people to live with a greater sense of ease and well-being, especially in situations where stress and trauma are unavoidable and inescapable.

## Methods

### Study design

This was a qualitative descriptive study that investigated stakeholders’ subjective perceptions and experiences [24] of MBP adaptation within low socio-economic settings, with the intention of offering a deeper understanding of the research topic and a more defined protocol for adaption [25, 26].

### Setting

The study was conducted in a low socioeconomic context in Cape Town, South Africa. Although the apartheid state was replaced by a new democratic government in 1994, the South African population continues to face many socioeconomic challenges, such as high unemployment rates, poverty, social inequality and inadequate access to public services [2]. As assessed in 2022, inequality remains among the highest in the world, and poverty is estimated to be at 62.6% of the national population [27]. The city of Cape Town in particular, is characterized by a vast disparity between well-off and poor communities as a legacy of the apartheid-era Group Areas Act and forced removals that separated families and communities. The consequent breakdown in family and community relationships has resulted in high levels of violence, crime, gangsterism, and poverty [28]. Furthermore, life in a low socioeconomic setting, such as that outlined above, is associated with high levels of chronic stress and compromised health and well-being [29].

The core setting for the study was The Big Issue (TBI) print magazine, which is based in the central Cape Town suburb of Woodstock. TBI was launched in Cape Town in 1997 to offer income to unemployed and marginalized individuals – the magazine vendors – living in low socioeconomic settings. Vendors purchase the magazine from the TBI head office and then sell it in public spaces around greater Cape Town for a set amount, making 100% profit from each sale [30]. After exploring several alternative settings, TBI was selected as the setting of this study due to the accessibility of participants for the research.

### Participants: population, sample size and sampling

Purposive [31] and snowball [32] sampling were used to select eligible participants. The inclusion criteria were: age 18 years or over, an understanding of mindfulness and sociocultural adaptation in low-resource settings, an understanding of the TBI vendor community and its needs, or potential future beneficiaries of an adapted MBP. A total of 14 participants were interviewed.

TBI was approached via an email to the managing director, and the proposed study was introduced. The director was enthusiastic about the research and initiated the process of contacting and interviewing the staff and the vendors. Two members of the TBI staff were purposively selected [31] based on their knowledge of the vendors’ needs. Another two were selected by snowball sampling [32] following the first author’s request for any other member of the TBI team who would be suitable for interviews. The first author sent emails to TBI staff outlining the study and inviting their participation. Following their agreement to participate, informed consent forms were sent to all TBI staff. Signed copies were returned to the first author prior to the interview.

Before vendor selection, the first author conducted a 30-minute pre-interview mindfulness-based workshop with vendors at their monthly meeting at the TBI head office in Woodstock, Cape Town. TBI staff suggested that the best approach for this study was to initially offer a 30-minute mindfulness workshop as part of the monthly vendor meeting, which regularly incorporates workshops on a variety of topics. The workshop offered an explanation and experience of mindfulness practice, as well as an opportunity for vendors to reflect on their experience of the practice. It also offered a brief explanation of the study, followed by an invitation to vendors to be interviewed for the study. Only four vendors volunteered to be interviewed. All four were presented with informed consent forms prior to being interviewed. The content of the forms was verbally outlined before the vendors signed. This process was supervised by TBI staff.

The workshop aimed to encourage vendors to volunteer to be interviewed by introducing them to mindfulness and its potential benefits, informing them about the research and familiarizing them with the first author so that they might be more open to sharing their viewpoints. Only three TBI vendors who had free time volunteered to be interviewed. When these vendors were asked if they knew other vendors who might be willing to be interviewed, none offered any suggestions. A further vendor subsequently volunteered and was independently interviewed.

The six mindfulness professionals were purposively selected [31] based on the first author’s knowledge of experienced and appropriately trained facilitators running MBPs within the greater Cape Town area. As these mindfulness professionals already knew the first author, they were approached directly via email to introduce them to the study and to invite them to participate.

### Data collection

Data were collected via semi-structured face-to-face interviews (*n* = 3), online interviews (*n* = 6) and two focus groups, one in person with vendors (*n* = 3), and one online with TBI staff (*n* = 2), ranging from 30 to 90 min, depending on the interviewees’ available time. The lead author conducted all interviews. The interviewees chose the times and places of the interviews.

TBI vendors live around greater Cape Town and use the TBI Woodstock office as a central communication point. It was, therefore, most convenient for the focus group interview with the vendors to be held at the TBI office after their monthly meeting. One vendor was interviewed at a coffee shop near their work pitch. Two TBI staff members were interviewed in person at the TBI office, and an online focus group interview with the other two staff members was conducted via Google Meet. The interviews with the mindfulness professionals were conducted online via Google Meet. All interviews were conducted in English by the first author, between July 2023 and March 2024. By the time of the last two interviews, data saturation was achieved, as no new themes had emerged.

Three interview questions were prepared, one for each group of participants (Table 1). Each question was drawn from the first author’s experience of working in the field of mindfulness and within low socio-economic settings and was phrased to be applicable to the group of interviewees. The interview questions were developed in consultation with the third author, who is experienced in advanced interviewing techniques and question design. The interview question for each group was used for both individual interviews and focus groups.

As TBI only has four staff members who work directly with the vendors, and all four agreed to be interviewed for the study, this question was not piloted. Gaining access to the vendors was complex and difficult, so the question for the vendors was also not piloted, especially as only four vendors volunteered. As a larger number of mindfulness professionals were available to be interviewed, it was possible to pilot the question. The pilot interview was conducted online, with the question eliciting responses that were appropriate for the research. The pilot was thus deemed acceptable, and the question was used for the other participants in that group. The data from the pilot interview were included in the overall analysis along with the data from the other interviews. The interview guides, questions posed, and data collection procedures remained consistent throughout study.

Each interview started with an invitation for the participant to offer relevant details about their life and work, followed by a core interview question based on the lead author’s knowledge of mindfulness and experience in facilitating MBPs within various settings. The research question posed to each group differed according to what was appropriate for that group. Following the interviewees’ responses, probing was used to encourage further clarification and expand on their original reply [33].

**Table 1:** Interview questions

GROUP	QUESTION
Mindfulness professionals	*From your experience of working with mindfulness within low socioeconomic settings*,* what have you learned about adapting these initiatives to be of greater benefit to the participants?*
The Big Issue (TBI) staff	*From your experience of working with the TBI community*,* please could you share your thoughts on how an MBP oriented toward enhancing wellbeing could be tailored to be of most benefit to the participants*,* i.e. what factors should be considered when adapting and implementing an intervention in this community?*
TBI vendors	*Vendors were offered two questions that were clear and direct*: *What would make it more likely for you to choose to join a program?* *What would make it difficult for you to attend a program?*

(For full interview questions, see Supplementary File 1)

All the participants agreed that the interviews be audio-recorded. After the interviews, the verbatim transcriptions were checked and imported into Atlas.ti for coding and analysis.

### Data coding and analysis

The first and third authors (SF and TH) independently familiarized themselves with the data by rereading all the transcripts. The first author coded and thematically analyzed the transcripts according to Braun and Clarke’s 15-point checklist for thematic analysis [34]. The initial codes were developed inductively during the reading of the transcripts, which allowed the themes to build out of the data rather than attempting to organize the data into a-priori themes. During the subsequent analysis, codes could be reassigned, and additional codes could be added.

### Trustworthiness

To safeguard the trustworthiness of the study, strategies were implemented to enhance the credibility, dependability, confirmability, transferability, and authenticity of the research [35] (see Supplementary File 2). Throughout the study, ethical considerations such as permission to conduct the study, confidentiality, respect, human dignity, beneficence and justice were adhered to.

The trustworthiness of the study was grounded in the lead author’s background. She holds an honors degree in psychology, with postgraduate diplomas in adult education and the facilitation of mindfulness-based interventions. Her work and previous research focuses on low socioeconomic settings, including researching strategies for quality schooling in low-resource schools and as a facilitator of mindfulness-informed parenting programs in a low-resource community in Cape Town.

Further strategies were followed to strengthen the study’s rigour. The third author offered oversight of the process of coding, analysis and interpretation. All the mindfulness professionals who agreed to participate in the study were well-known to the researcher, which made for open and honest discussion around the programs they had facilitated and which would serve to offset positive bias. Pre-interview meetings were held with all but one TBI staff member to establish a sense of ease and for interviews to follow an informal structure of a “chat” or “conversation”. A similar approach was assumed with the vendors, except the researcher ran a half-hour mindfulness-based stress management workshop for the vendors instead of individual pre-interview meetings. The workshop’s intention was not only for the vendors to learn a little about mindfulness but also to feel comfortable in the researcher’s presence so that they might be more open to sharing during the interview.

All interviews were conducted according to the attitudes of mindfulness [8]. Specifically, trust was established before the interviews, and the discussions were grounded in nonjudgment, acceptance, patience, and curiosity. In addition to the above, the research was conducted and written up according to the 32-point Consolidated Criteria for Reporting Qualitative Research (COREQ) checklist to increase trustworthiness [36] (see Supplementary File 3), as well as the Braun and Clark 15-point checklist (see Supplementary File 4).

## Findings

Fourteen stakeholders offered insights into the adaptation of an MBP for TBI vendors: (1) six mindfulness professionals with expertise in mindfulness, most of whom regularly facilitate MBPs in low socioeconomic communities in Cape Town; (2) four TBI staff members who understand the needs and challenges faced by the vendors and who regularly arrange or run supportive programs for the vendors (See Table 2); and (3) four TBI vendors. The researcher noticed a reticence on the part of the vendors to volunteer for the study. The vendors indicated that they wanted to participate, however they needed to get back to selling the magazine, which is fundamental to their income (See Table 3).

**Table 2:** The demographic characteristics of the big issue staff and mindfulness professionals

Interviewee	Level of education	Field of work	Years of experience (5-year range)	Detailed description of work
TBI-1	Diploma	Office manager and administrator	15–20 years	Organizing workshops and catering for vendors; doing field visits to vendors’ homes; co-ordinating Saturday school for vendors’ children; communication channel and translation services for vendors
TBI-2	BA Hons	Leadership coaching/HR consulting/ training	20–25 years	Runs a coaching consultancy and is a part-time consultant for the TBI and other small businesses on all HR issues.
TBI-3	BSocSci	Social work	1–5 years	Social work intern at TBI, employed 2 days per week to support vendors
TBI-4	Gr 12	Training and research	20–25 years	Runs monthly 2-hour training sessions at TBI for the vendors, using active and participatory learning models. Training focuses on life skills, wellness, chronic illness and sexuality.
MP-1	BA Hons (Psych)	Trauma and grief counsellor; MBSR teacher; TRE facilitator	20–25 years	Private practice, consulting for NPOs, running courses, training, and offering supervision. Facilitates the MBSR program.
MP-2	MBChB/PhD	Medical practitioner/ psychotherapist/ mindfulness teacher, trainer and supervisor	25–30 years	Private psychotherapy practice in Cape Town; developer and program coordinator of training in mindfulness-based interventions; MBSR facilitator for 20 years.
MP-3	BSocSci Hons (Psych)	Social worker; grief counsellor; MBSR facilitator; coach	20–25 years	Private practice, consulting, and contractual work in social services, grief support, and mindfulness.
MP-4	Gr 12	Mindfulness practitioner/ facilitator and life coach	5–10 years	Works as a life coach with youth from Cape Town townships toward personal development, using modalities such as MBSR and Non-Violent Communication. Currently facilitating MBSR and offering career coaching.
MP-5	BSc Hons	Mindfulness for children and adults in low and medium socioeconomic settings	5–10 years	Currently facilitating mindfulness with adults at NPOs in Langa, Cape Town.
MP-6	BBusSci	Youth development	5–10 years	Psychosocial support and mindfulness training for under-resourced youth. Freelance consulting.

*TBI* The Big Issue staff, *MP* Mindfulness professional, *NPO* Nonprofit organization, *MBSR* Mindfulness-based Stress Reduction, *TRE* Tension and Trauma Releasing Exercises, *HR* Human Resources

**Table 3:** Summary of vendor stakeholder demographics

Vendor	Area of residence	Home languages	Highest level of education	Time as vendor (range)	No. of dependents (range)
V1	Stellenbosch	Xhosa	Gr 12	Less than 1 year	1–3 children
V2	Khayelitsha	Xhosa and English	Gr 11	1–5 years	4–6 children
V3	Lamazwe township	Xhosa and Afrikaans	Gr 12	15–20 years	4–6 adult children and grandchildren
V4	Gugulethu	Xhosa	Gr 10	10–15 years	4–6 children

**Fig. 1:**
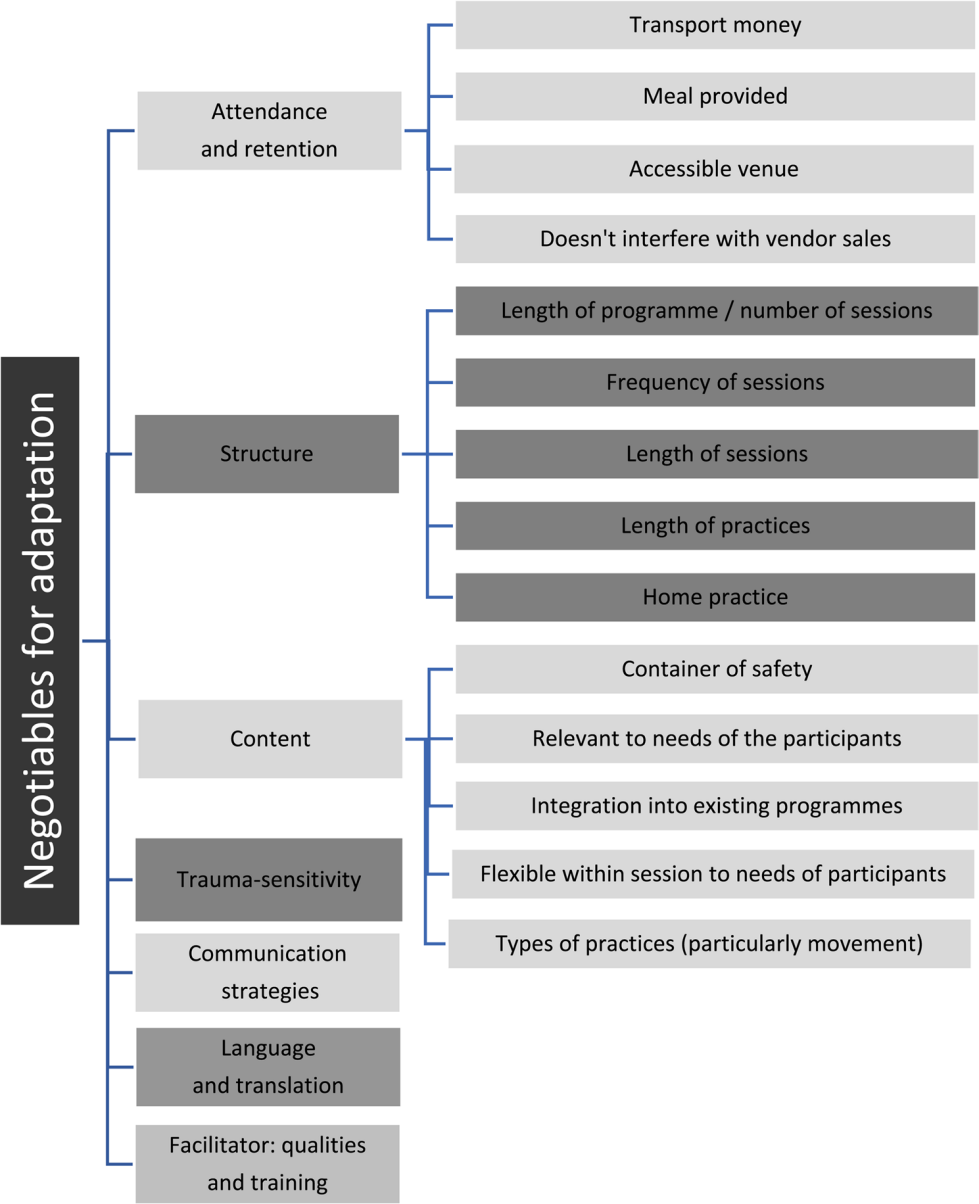
Thematic map demonstrating the negotiable elements for adapting a mindfulness-based program

Seven themes were identified in the data that inform the negotiable elements of adapting an MBP in a low socioeconomic community: initial and continued attendance; program structure; program content; trauma sensitivity and awareness; communication strategies, including how language is used and the possible need for translation; and the training and personal qualities of program facilitators. The seven themes were each informed by several subthemes, as shown in Fig. 1.

### Attendance and retention

In any setting, the factors that encourage initial and ongoing attendance of MBPs should be considered. The vendors indicated their appreciation for an MBP but clearly stated that they would only attend if there were no personal costs, such as transport. They were also more likely to attend if offered a meal at each session.

The problem is that … the transport money. It’s costing us a lot … As long as you gonna attend the program. But if it’s gonna offer you food, transport money … [that’s] gonna help. (V4)

Lunch. That’s very important to attend the program. I don’t have a problem with that. And then the transport money as well. (V3)

TBI staff reiterated the importance of incentivizing vendors to encourage attendance by commenting that “last year the attendance was quite great because we offered them transport … but then this year … attendance hasn’t been so great … because of the fact that we haven’t been giving them transport money”. (TBI-3)

Vendors suggested that venues should be accessible for convenience and safety. Although they sell the magazines at their pitches across the Cape Peninsula, the TBI office in Woodstock is a central point where they all meet regularly. By meeting at the office, they will not incur any additional travel time or costs.

I think here [TBI office] it is the nearest because after we finish here, we can go to Cape Town, and here all the vendors can meet whether they come from different places. This is the safest place. (V3)

The mindfulness professionals also mentioned that the venue should be considered when adapting a program.

So if I look at how I’ve adapted things, practical things … would be holding the sessions in a place where people can easily get to. (MP-5)

Participants stated that the MBP should not result in loss of sales and essential income. Vendors suggested that attendance would be better during quieter sales times, such as in the winter or after the summer holiday. Over peak sales times, however, attendance might be less likely:

You see, you sitting there, you attend the workshop meeting. When you go back to your pitch you lost a lot of customers … I don’t mind about in June, July or the beginning of the year, I don’t mind … the problem is that now it’s the season, it’s December holiday. Gonna be making a lot of sales. (V4)

The above was reiterated by TBI staff, who noted the following:

… we’ll have to invite them to come to the office and do the practice here, you know, as long as it doesn’t interfere with their sales because they are a sales-driven group. (TBI-1)

### Structure

The term “structure” refers to design elements of the MBP, including duration, frequency and number of sessions, lengths of sessions and practices, and the inclusion of home practice. These elements hold the content together and provide the scaffolding within which the program’s content may be presented.

The vendors offered a sense of what would be feasible in terms of frequency and length of sessions:

Like in vendor meetings, once a month ‘mos’ [of course]. (V2)

If it can come once a month, it can be helpful for us as vendors. (V1)

Just one hour’s right. Not more…. (V4)

When asked about the feasibility of running an 8-week program, TBI staff responded that “it’s too much” (TBI-4) and that it “would be very difficult” (TBI-2). This was reiterated by the mindfulness professionals, who, in their experience, also found that “you can’t go in there with an eight-week program … for our communities, it’s rare that you’re actually going to get that commitment from where people are going to be consistent because of the fact that they don’t have a lot of time” (MP-3). Workshops were suggested as an alternative “every quarter … or just for one day … but this every week for an eight-week period, it doesn’t necessarily work” (MP-3).

One TBI staff member suggested expanding an MBP with longer breaks between sessions, which indicated that “it will work only when there is a vendor meeting day … that would be once a month. Maybe like 30 minutes, once a month. Then that would work” (TBI-1). Participants suggested that the MBP should comprise fewer and shorter sessions, increasing feasibility and improving attendance.

Regarding the duration of sessions, the vendors did not offer any suggestions, as they were unfamiliar with mindfulness. However, one TBI staff member suggested “keeping it shorter. But enough for them to really feel those benefits” (TBI-2).

The mindfulness professionals explained that, in their experience, offering shorter practices is more manageable for MBP participants:

Duration of practice is a major factor that does need to be adapted. (MP-2)

It’s always like shorter versions of each of the kind of MBSR-typical practices. (MP-6)

While most of the mindfulness professionals had worked in low-resource settings before, their experience in these settings was not necessarily relevant to the specific TBI setting. For example, one of the professionals who ran adapted MBSR programs in an NPO in Langa suggested that “I only do two hours … each session is two hours, instead of the normal, two and a half to three hours” and that “the practices in the sessions are the same as per the MBSR curriculum” (MP-5). While this may not be feasible for TBI vendors, it may be worth considering that standard MBSR practices and structure might be effective within different low-resource settings.

When asked about home practices, the TBI staff expressed that it was unlikely that vendors would engage in mindfulness practices at home:

No, they won’t have time, because from the pitch they have to go back home. There are kids, preparing for supper. There’s TV, there’s being tired, sleeping, preparing for tomorrow. There’s a lot. They won’t be able to do it at home, I’m telling you. So it should be done…at the session, here. (TBI-4)

The mindfulness professionals were, however, regularly setting home practices for their group participants, even though this was substantially reduced from those suggested in a conventional MBSR program:

I offer a lot more flexibility … I actually give them a 15-minute recording in addition to a 40-minute recording so that they can see what works best for them. (MP-5)

We have to ask is it more important: that you do a 10-minute body scan every day or every second day, or one forty-minute body scan once every three weeks? (MP-2)

One of the mindfulness professionals suggested that a group home practice might be achievable, which could be considered for the vendors:

You could encourage them to continue the practices together, and it’s not so isolated … it sort of makes it easier for them … it sort of just changes the dynamics of the system if it’s like a communal thing. (MP-3)

One of the main concerns for TBI vendors is that their low earnings preclude most of them from affording smartphones. Even if they do own smartphones, accessing free Wi-Fi where they can stream recordings is not straightforward. In other settings, the mindfulness professionals indicated that “[I] have it all downloaded and I will send it to them week by week” (MP-1) or that “[I] actually put it on Google Drive and then they stream it from Google Drive … At the organizations where I work, there is Wi-Fi, so they could download it when they at work and then have it on their phone” (MP-5). However, the first author’s experience communicating with vendors via phone, including WhatsApp, suggests that this approach might not be feasible for the TBI vendor community.

A further structural element of an MBSR program is the full-day retreat between weeks six and seven. This retreat element was raised by one mindfulness professional, who indicated that she adapted this element as follows:

What I normally do, there is a morning, so it’s more a four- to five-hour retreat. (MP-5)

One TBI staff member offered a similar suggestion and suggested that occasional vendor meetings could be dedicated “mindfulness days.” This “mindfulness day” could be held during the monthly vendor meeting time, but none of the other regular presenters, such as the art teacher or nutritionist, would be invited so that the time could be focused purely on mindfulness:

We let the vendors know that it’s gonna be a mindfulness day for that day. Then on that day, we will have not to invite other stakeholders on that day. (TBI-1)

This is in keeping with the suggestion of a mindfulness professional to offer “workshops … it’s one day that [they] can go to” (MP-3). One TBI staff member expressed the structuring as “this has been something new today … all of the logistics behind the times, the place and all of these things. So it’s all about how we going to give this idea to them and how we going to make sure that they understand it” (TBI-3). Any MBP would be a new experience for TBI vendors, the staff and facilitators, and several iterations might be needed before achieving a feasible and acceptable format.

### Content

Content refers to the material covered within the structure of the program. The content encompasses the nonnegotiable and negotiable elements of mindfulness [7], which may be adapted to a particular setting and participants’ needs.

#### Container of safety

The most important subtheme regarding content is the creation of a container of safety: a supportive, nourishing, kind and nonjudgmental environment where participants can feel safe to hold, explore and share both their inner and outer experiences during the program. This theme is closely connected with the theme of facilitator qualities, as it is often the attributes and presence of the facilitator themselves rather than any specific mechanisms, that create a sense of safety in the group.

Among mindfulness professionals, creating a container of safety was seen as “the bricks and mortar itself of mindfulness” (MP-2). While this holding environment may be a nonnegotiable element of any MBP, it was suggested that “the degree to which you adapt … needs a different … appreciation and deliberativeness in our context than it does in upstate New York or in London or in Copenhagen or Sydney.” (MP-2). In different contexts, creating a safe container might include “agreements around confidentiality” (MP-5), reading “poetry: that has been helpful because … that talks to all of them” (MP-4), or lighting “a candle for all of us gathering in the present moment together and especially those who maybe couldn’t make it that day. And I think that those symbolic gestures are very important because it kind of gave everybody a sense of “I’m seen” without us having to say another thing” (MP-1). Creating a sense of containment could include caring for the immediate needs of the participants:

I’ve brought food for the beginning of a session … And I found that it just creates a sense of being welcomed … when she arrives at the session, she’s pretty tired. So maybe a cup of tea or coffee is gonna help her to feel, you know, a little bit ‘okay, cool. Now, this is a space where [I can] stop and be’. (MP-5)

Most mindfulness professionals mentioned that building a “safe container” required ensuring that “the attitudes of mindfulness are centre stage. Because, as you mention those words to people in any socioeconomic group anywhere, those words are life-giving. Beginner’s mind, loving kindness, trust, letting go, nonjudgment, nonstriving, just good effort … I often use the attitudes as the sort of bowl that holds it all.” (MP-1). They spoke to the importance “of trust-building beforehand, … with maybe humor or like getting people ready and allowing a lot of time for people to get to know each other and setting the ground rules of like how we’re gonna engage with each other …. Just allowing space that people can adjust to being in the space together… before you start teaching any mindfulness” (MP-6). The container of safety that holds the group depends on the qualities and capacity of the facilitator – which are capacities that may be cultivated – to embody welcome, trust and safety.

Having a welcoming attitude and a welcoming space and a humbleness is what allows people to come back. (MP-5)

Hold the space there and acknowledge them all … It slows them down, and it also makes them feel safe around me and in the space and when I’m around. (MP-4)

It’s got to feel safe. (MP-1)

While not offering specific guidelines, TBI staff mentioned that “having people who [the vendors] are familiar with … increases the trust factor and their openness” (TBI-2) and that “just slowly building that relationship and then they would definitely look forward to your sessions and welcome you” (TBI-3). The professionals noted that facilitators who come from outside the community should be sensitive to the context of the MBP:

I would say don’t just go into a community … it’s good to get to know the people from that community, um, because there’s a risk of being seen as, yeah, the dominant person, especially if you don’t come from the same socioeconomic status. (MP-4)

#### Relevance to participants

The content of an MBP should also be relevant to the vendors. TBI staff suggested that “maybe you first do a needs assessment” (TBI-4) so that the program could be “a tailored one for this particular group.” They noted that “this is for them, you know, for their own benefits … It is nothing to do with us imposing it on them … It’s best to ask the vendors themselves what they want” (TBI-3). “It should come from them” (TBI-4).

The mindfulness professionals spoke about how “the value of adapting the program is so that it speaks to [participants] … so that it fits into their lives” (MP-5):

We need to adapt, to make it accessible and also relevant to the context that they are living in … that it can be something that they can actually relate to also … we need to be aware of the psychosocial issues of that specific community. (MP-3)

It would need to emerge out of a conversation from within the community … and also to have a conversation about some of the specifics of the challenges that that community is facing … Ideally, you want the program to arise from within the community because then … [it is] more likely to be successful. (MP-2)

One mindfulness professional suggested that there “needs to be some kind of element … that can actually address some of the issues that they experience … that they struggling with” (MP-3). The practices included in MBPs should be adapted to reflect participants’ needs.

The relevance of an MBP for vendors might be improved by integrating the program into existing offerings that meet their needs, such as health or nutrition programs, which are regularly run by the TBI:

I think it’s a nice way in … finding something that will slot in possibly with programs we already are running would be helpful. (TBI-2)

TBI staff members suggested that “sales training is what they would grab with both hands because it helps them with their sales out there: how to behave and what to expect and what not to … you know” (TBI-4). A further suggestion was that mindfulness could be included in programs that develop “the awareness of other things … like the HIV workshops” (TBI-1).

The vendors suggested programs that are underpinned by mindfulness, but that also address their other needs, such as finances and domestic violence:

Maybe I think about how to save the money … I love that one. (V4)

This program is very important, especially for males … All I can say is that females are in a abused relationship mostly. So this is helpful for them, and also for us as males. (V1)

#### Integration into existing programs

Mindfulness professionals spoke to the effectiveness of weaving mindfulness practices into existing programs. For example, a facilitator involved in grief work noted, “I’ll be doing a workshop on grief, but I will start it and hold it and teach the mindfulness principles as we start into the other work … I’m also going to be teaching you how to come to awareness, notice what’s here. It all frames it.” (MP-1). Another example was how the attitudes of mindfulness could be woven into the Trauma and Tension Release Exercises (TRE) work they do with clients:

When you’re doing TRE … you’re coming to awareness, but you’re always settling, coming to presence and grounding first … mindfulness helps them get to the TRE (MP-1).

A mindfulness professional involved in training young software developers from low resource communities integrated mindfulness into participants’ daily reflections on the technical curriculum they were working on. They started with “the informal practices of saying: okay, before we start today, can we just pause and notice. Like just introducing this language of pausing and noticing the ground and the chair” (MP-4).

Another professional stated that they, “incorporate mindfulness into self-care workshops with other social workers” (MP-3). They experienced that integrating mindfulness into an existing program afforded participants emotional relief by offering them the “time to touch into what’s happening in their bodies, within their hearts, within their minds … they wish they could stay there in that space” (MP-3).

#### Flexibility

Mindfulness professionals suggested that facilitators should be flexible in their ability to adapt the program to meet the participants’ needs and be skilled and agile enough to adapt to these emerging needs individually and within the group. This subtheme is closely related to the theme of facilitator qualities and training.

Participants felt that it was valuable to “come with an idea or a structure which is flexible and fluid and got integral elements” (MP-2), and then “in feedback with the participants, we might realize that adaptations need to be made” (MP-2). The facilitator should be “open to being flexible if something isn’t working to changing it and being open to some feedback. But not being so open to feedback that the whole thing just starts to feel uncontained” (MP-2).

In a field where present-moment awareness is fundamental, the ability of the facilitator to adapt to what emerges in the moment, “to resonate and work with where they are” (MP-2), is essential. The value of this was expressed by one of the mindfulness professionals, who noted that “life in the groups I work with happens day to day. In fact, people do live very much in the present, so there’s not a lot of planning for the future, so I adapt to where the people are at” (MP-5).

Flexibility also encompasses being culturally sensitive and being able to adapt to the context, especially when working in a community as an outsider. It might be impossible to know in advance how to adapt the program to a specific setting, so being open and flexible to the requirements of the context is important:

I find that in the area where I work, there’s a lot of fluidity … there’s a lot of shifting … But it’s really meeting your participants where they are at … coming in with a humble heart … being open to learn from them. (MP-5)

The ability to respond flexibly to circumstantial events is also important. For example, “life is so chaotic often … people do arrive late, and that is just how it is. A lot of the people are using public transport, and it’s not within their control often to get there exactly on time … and, you know, I had one participant who, um, like, two days before their house had burned down … So I’m a lot more flexible … sensitive to the continuous daily trauma that people are experiencing.” (MP-5).

One mindfulness professional shared an anecdote supporting the importance of flexibility in unusual circumstances. During an individual phone-based practice with a participant, the facilitator “asked the lady ‘where are you now?’ And she said, ‘I’m sitting on the toilet now. It’s the only quiet space. You keep on talking. I’m listening to you. Keep on!” (MP-3).

Being flexible to the group and individual needs while balancing the nonnegotiable elements of an MBP alongside a robust container of safety was succinctly expressed by a mindfulness professional:

You’re living in the present moment, you’re aware people are coming in with all their things, and you’re moving with the cloud of it … You’ve got some structure to it, but at the same time, you’re adapting as you go … That’s important – to be able to be flexible. (MP-1)

#### Types of practices

When adapting an MBP, the types of practices offered, and how they are offered, should be considered. Therefore, rather than saying, “It matters what you practice, I think it would be more accurate to say it matters that you practice” (MP-2).

One professional noted that Kabat-Zinn recommended that MBSR participants “practice them all … and see what works for you”, but also commented that “we don’t know yet whether that’s a wise approach in a low resource environment … And in a trauma-dense environment, we don’t know the answer to that question” (MP-2).

The interviews revealed that many mindfulness professionals are offering standard, albeit shortened, MBSR practices to their groups in low resource settings, and that these practices are well received:

I do the traditional body scan … that’s not any different … Sitting practice, yeah, it’s the same so that there’s not much change in that. (MP-5)

We do a lot of … the body scan, breathing … the body scan is very powerful … the loving-kindness meditation works very well. (MP-3).

A lot of my practices are typical mindfulness practices like when it’s breath meditation, standing meditation, and walking meditation … those practices are the most effective for me because they’re simple and they’re like consistent and they’re clear, and they can be compassionate and kind. (MP-6)

One professional noted that naming a practice, for example, an “awareness of breath exercise,” felt too formal and intimidated their group of young adults. Instead, without naming it, “[I] try as much as I can to make it very accessible like just noticing your feet on the floor, noticing your in-breath and your out-breath and just noticing how it’s like to be here right now … without focusing on okay we are doing an awareness of breath exercise” (MP-4).

Mindfulness professionals and TBI staff mentioned that movement practices were particularly well accepted in their groups, regardless of the form in which they were offered. Professionals mostly used adapted yoga practices and stretches, which were well accepted by group participants:

I think even if it’s just the basic stretching … even if it’s just the mountain pose.(MP-3)

I’ll stop at a certain point and say, listen, let’s just do some stretches. (MP-1)

They ask about it. ‘Are we doing yoga? … It’s something they look forward to. (MP-4)

One mindfulness professional worked at an NGO where “most of the people are Xhosa speaking and love dancing … a lot of them are musicians” (MP-5), so they adapted the movement practices to be more culturally accessible:

The mindful movement is a lot more fluid than the kind of traditional poses, the yoga poses from the MBSR … I just try to make it more African in a way. (MP-5)

According to TBI staff, it was important that, beyond having the skills to guide movement practices, the facilitator has the capacity to resonate, attune and “be with” participants:

You need to be down on the earth with them; you don’t even need to come and say now we’re gonna be doing yoga or take your chair … or the dog … you need like show them. Be also like involved in what you want them to do. Do it first, and then they will do it after you. (TBI-1)

Interestingly, the mindfulness professionals lit up when speaking about movement practices and became energized and excited – almost as if simply talking about an embodiment practice brought them energy and joy. In describing their group participants’ responses to the movement practices, they used the word “love” several times.

It’s the first time that people have ever engaged in something like that. But they love it. They do love it … They love the mindful movement.” (MP-3).

I definitely bring in the movement … Oh, they love it! … right across the board, they love it. Always. (MP-1)

### Trauma sensitivity

Trauma sensitivity is an important consideration when adapting an MBP. Life in low-resource contexts in South Africa is characterized by trauma, particularly in Cape Town’s townships, which were shaped by the legacy of apartheid and especially forced removal, including poverty, gangsterism, violence, alcoholism, and drug abuse. The pre-interview workshop with the vendors offered insight into the spectrum of this trauma, with vendors naming finances, homelessness, poor sales, sickness, transport and challenges with children as some of the difficulties they experienced. While some vendors eased their struggles by singing and dancing, most named the following coping strategies: ignoring, sleeping, eating, taking tablets, drinking alcohol, smoking marijuana, and hitting their children. Although this data from the pre-interview workshop was not included in the study, it reflects what was shared in the participant interviews.

The TBI staff and the mindfulness professionals confirmed the vendors’ experiences of trauma. MBP participants from low socioeconomic contexts are very likely to arrive at the program with some degree of traumatic stress:

If you’re gonna say, you know, you can’t come if you’ve experienced trauma in the last six months… you’re not gonna have any participants. (MP-5)

In the South African context, if you want to introduce mindfulness … you need to address the psychosocial issues that they face on a daily basis. (MP-3)

The mindfulness professionals suggested that it was important to be “sensitive to continuous daily trauma that people are experiencing … and adapt according to that” (MP-5) rather than presenting a preconceived program. In addition, they suggested that “when people are traumatized, [the practices] need to be much shorter” (MP-1). The window of tolerance of traumatized participants is much narrower, and “I just don’t think there’s the bandwidth to be able to do these long elongated practices” (MP-1) of a standard MBSR program.

The formal elements of mindfulness should be gradually introduced. One mindfulness professional felt that a full MBSR was too intense, initially, for groups at a training college for low-resource youth. A more acceptable approach was to offer “noticing sessions [that] would be really brief, like a couple of minutes, and then as time goes like we’ll just do five minutes … just sitting, noticing the breath or noticing the body and the breath, with our eyes closed, or with our eyes open … so by the time we start doing the formal practice, then, yeah, the practice could be anything from 10 to 20 minutes” (MP-4). In trauma-dense settings, slowly and gently introducing mindfulness practice can be effective before moving on to more challenging, in-depth MBSR practices.

Another professional supported the slow introduction of mindfulness to develop the capacity “to be still. To be still is enough. So we just do the titration thing where we just do what you can, just touch it” (MP-1). This gradual introduction in a safe, kind and accepting environment is fundamental in situations such as that described by an interviewee, where “a lot of women in the room had been raped somewhere along their life. A lot of almost everybody had encountered severe violence. People have watched their relatives being killed in front of them. So … you are holding a situation very gently, and I think people respond most to the fact that they’re respected, they’re safe, and there’s loving-kindness in the teaching” (MP-1). In trauma-dense contexts, simple practices are supportive:

It’s around coming to notice, coming to settle, bringing presence, present moment awareness, it’s self-compassion. (MP-1)

One practitioner related that “a wise approach in a low-resource environment … in a trauma-dense environment remains to be fully understood…. We don’t know the answer to that question” (MP-2). Until we have a deeper understanding, it is best to “go on this assumption that [trauma] was high and work backwards from there” (MP-2), starting with straightforward, short practices grounded in both contextual awareness and kindness and building from that according to the capacity of the participants.

### Communication

Communication refers to the strategies used to share information with MBP participants, which should be adapted to ensure that all participants receive program information.

While mindfulness professionals found WhatsApp and Google Drive convenient for communicating with their groups, including sharing information such as recorded practices and documents, many TBI vendors do not have reliable access to these options. A TBI staff member noted that, generally, with few exceptions, “they don’t have smartphones” (TBI-1).

One of the vendors mentioned that “WhatsApp” was feasible and that “TBI had a WhatsApp group for the vendors” (V4). However, the first author’s experience was that vendors could not easily or regularly receive or send messages online and could not afford airtime. Communication was a challenge.

While one vendor indicated that receiving reading material or written practices might be feasible, a TBI staff member mentioned that “even with the reading material, someone needs to intervene because some of them cannot even read and write … Pictures would be better for them to understand” (TBI-1).

Before starting an MBP, a feasible and acceptable communication system with the participants should be established. In under-resourced communities, such as TBI vendors, communication strategies will look very different from conventional messaging for mainstream MBSR participants via WhatsApp or email. Any online elements would certainly not be possible.

### Language and translation

For MBPs in low socioeconomic settings, delivery language and the need for translation should be considered. All the vendors interviewed indicated that their mother language was Xhosa. While one of these vendors felt confident they could manage if an MBP were run in English, TBI staff suggested that translation at some level would be essential. TBI staff confirmed that “the majority of our vendors are Xhosa-speaking. We’ve got white, we’ve got coloreds, but the majority is Africans, whereby we speak Xhosa, Zulu and sometimes Sotho” (TBI-1). TBI staff also suggested that if facilitating in English, “you would need to keep it very basic language…making it understandable, even practical” (TBI-1), but alongside this, “translation will play a huge part” (TBI-3).

The mindfulness professionals had various opinions on the importance of delivery language and translation. One of the professionals expressed that:

… just to change or use a different language is incredibly significant … What is lost in translation? What is the effect on the felt sense of receptivity in the mother tongue, for example? And what happens if I’m teaching somebody or a group of people that I don’t have access to that mother tongue. (MP-2)

Other professionals indicated that in their groups within low socioeconomic communities, the use of English was not perceived as problematic, even if most of the participants were first-language Xhosa-speaking. They indicated some valuable strategies for accommodating potential barriers to understanding, including using simple language, speaking slowly and inviting other group members to translate informally:

I think it’s just to be sensitive to the fact that, you know, maybe not everybody knows the English word … keeping your language fairly not simple but like neutral … But I haven’t had somebody not benefit from it because of offering the course in English … And often there’ll be more than one person in the group that is able to translate if somebody’s not understanding … So I guess these are just kind of ways that I’ve adapted almost even unconsciously to make sure that, you know, everybody stays with with the group. (MP-5)

One mindfulness professional, whose mother tongue is Xhosa, prefers to facilitate in English, even with Xkosa-speaking groups, and will “try to find the simplest forms and sometimes I would modify … the language” (MP-4). This professional felt that “it’s very difficult to find the language in Xhosa … it can be really inaccessible. Xhosa … can be very difficult, even for me, and more so for these young people” (MP-4). They noted that mindfulness was challenging to communicate in the Xhosa idiom and that “there are also many Xhosa dialects which would make it even more difficult in a way” (MP-4) because not all the participants would understand the same dialect. It was, therefore, simpler to facilitate in English with translation to Xhosa where necessary.

### Facilitator qualities

While robust mindfulness facilitator training might be assumed as essential and ethically imperative for ensuring a pedagogically sound MBP, only one mindfulness professional mentioned facilitator training specifically. They noted that “training needs structure and it needs a well-defined process and it needs to have scaffoldings … How do we kind of communicate … that both the warp and the weft processes are necessary in the training, and in the offering that will come subsequent to the training where people share it. And how does the training itself equip somebody to do that? … What would it mean for a person post their training to go ‘I’m going to adapt’” (MP-2).

The mindfulness professionals and TBI staff did, however, speak to the importance of the qualities of the facilitator delivering the MBP.

Firstly, facilitators should be sensitive and attuned to the participants’ needs, within the context of the environment: I guess it’s not something you can really do training on if I can put it like that, but it’s more about having this open heart of what is best gonna serve this group of people … This is something in your heart. (MP-5)

Sometimes it’s like you do get training … but sometimes it comes within you. It’s one of the gifts that you have as a person sometimes. (TBI-1)

Secondly, participants mentioned that the qualities of facilitators, rather than any particular curricular element, were vital to an MBP.

The qualitative element is as important, like how one brings a kind of accepting and compassionate inquiry into people’s experience. (MP-2)

While the attitudes of mindfulness are nonnegotiable elements of an MBP, the interviewees named certain essential facilitator qualities if the program is to be well received by the group. These qualities are outlined in Table 4.

**Table 4:** Qualities of an embodied mindfulness facilitator

Trust	Having people that they are familiar with … that increases the trust factor. (TBI-2) Allow that relational time where trust is being built, and people feel like they’ve seen and accepted for who they are (MP-6)
Safety	I think one cannot underestimate safety and trust and respect (MP-1) They’re safe, and there’s loving-kindness in the teaching (MP-1) It also makes them feel safe … when I’m around (MP-4)
Respect	I think people responded most to the fact that they’re respected (MP-1) There’s a need to hold the space there and acknowledge them all (MP-4)
Welcoming	I think just really having a welcoming attitude (MP-5)
Curiosity	So curiosity, but like not curiosity in like sitting and watching, but curiosity and being with (MP-5)
Flexibility/responsiveness	Emergent flexibility … in relationship with what is arising in the present moment (MP-2) If that person is very flexible and not like a very strict person, they will interact and be with you. (TBI-1)
Humility	The humility of how you’re offering it (MP-1) It’s really meeting your participants where they are at and, coming in with a humble heart, being really just being open to learn from them (MP-5)
Kindness	And kindness, you know, towards yourself, towards others (MP-5) When you’re working with trauma, you can only work with kind eyes (MP-1)
Attunement	Understanding how people are in this country (MP-6) Ability to read the group (MP-1) You just pick up on subtle clues (MP-5) I even had to sort of go up and meet them where they were at. (MP-3)

Notably, the facilitator should feel an affinity for the group participants – particularly cultural and racial affinity – particularly as it may be “really challenging for people in communities to be taught by people outside of their communities with whom they don’t identify” (MP-2). When a facilitator or co-facilitator “from within the community [is] involved in the offering, [it] is more likely to be successful” (MP-2). There is less of “a risk of being seen as, yeah, the dominant person. Or especially if you don’t come from this same socioeconomic status” (MP-4). One professional commented that “the fact that I grew up in a township with like everybody else. You know that I can relate to like the thing: that makes me more similar to the people that I’m teaching, and that like helps the situation more than a technique that I have that I learned somewhere” (MP-6).

Thus, in respect of MBP adaptation, when establishing a training the selection of an appropriate facilitator is vital. The facilitator’s capacity to establish a robust container of safety, to attune to both the dynamic of the group and the needs of the individuals within it, and to be flexible and responsive to what arises moment-by-moment is fundamental. In addition, the facilitator should be in regular “supervision sitting in community, being in silence … where you can reflect on your practice … All of those are essential elements for anybody who’s going to be doing it in this context” (MP-2), so that their very presence embodies the teachings.

The first criteria is that you’ve done your own work before you offer anything. You’ve done your own personal work … it must be in the room by the fact that I’m doing it. And I think that the co-regulation of that is huge, because you can come in and a whole room can change by you just being steady, clear, you know, holding the practice. (MP-1)

If you’re not immersed in your practice, then better you don’t teach. (MP-2)

## Discussion

This study investigated the key themes and subthemes relating to elements of an MBP that could be adapted for the program to be more feasible within low socioeconomic contexts, in particular the TBI community in Cape Town. The discussion that follows highlights the nuances of adapting MBPs for communities affected by vulnerability and trauma, emphasizing the importance of facilitators in creating a safe and responsive environment. Facilitators should engage in continuous reflection and personal development to embody the teachings effectively. Communication strategies need to be tailored to the specific needs of participants. While mindfulness professionals find tools like WhatsApp and Google Drive convenient, there are not always accessible to all, particularly in under-resourced communities. Language and translation are significant concerns, especially in low socioeconomic settings where participants’ mother tongue, such as Xhosa, may not align with the delivery language.

Before offering MBPs in low-resource communities, program designers should establish the factors inviting initial and continued attendance in the target community. Key stakeholders should be involved in the adaptation process, which might vary across contexts.

In the TBI community, vendors indicated their willingness to attend any program offered by the organization, provided there were no personal costs, particularly if the MBP addressed their need for guidance with finances, parenting challenges and stress management. This finding is consistent with the literature indicating that attendance could be affected by financial constraints [3, 37, 38] inadequate childcare, lack of transport, conflict with work, health issues [3, 29], and unsafe housing and neighborhoods [3, 37].

Vendors indicated that they would be more likely to attend if offered transport money and meals, as long as the timing of the sessions did not compromise their magazine sales. Providing meals, transport solutions, financial incentives, and appropriately timed classes have been shown to encourage attendance and retention [3, 8, 29, 39–41].

The findings indicated that the structure of the MBP should align with the target community’s needs. If MBPs are offered at locations where participants meet regularly, such as schools, clinics or workplaces, then offering weekly or longer sessions might be feasible. The TBI community, however, is geographically widespread. Vendors work independently, selling the magazine in various locations throughout greater Cape Town. The standard MBSR format is not feasible in this context, as vendors have neither the time nor the funds to meet weekly at a central point. Both vendors and TBI staff indicated that incorporating an MBP into the monthly vendor meetings at the TBI headquarters would be most feasible. While studies referred to MBP settings, no studies have addressed the relevance of venue regarding attendance, retention, and the success of programs.

Within the context of TBI, a series of monthly meetings were seen as the most feasible approach. The TBI staff suggested occasional “mindfulness days,” perhaps once or twice a year, presented during the regular monthly meetings. Aside from essential business and administrative matters, the morning could be dedicated to learning and practicing mindfulness. The rest of the monthly meetings could accommodate an open-ended MBP with short practices integrated into the regular monthly meetings if this is well-received and beneficial to the vendors. For example, a mindfulness facilitator could be present to open and close the meeting with appropriate short practices, and mindful movement could be offered as a break between the agenda items. These short practices could be presented in a way that vendors could use them as supportive home practices. A pilot program accommodating these approaches should be run and assessed for effectiveness.

Literature suggests that MBPs run in low-resource settings vary substantially in terms of the number of sessions, length of sessions and length of practice and that, in many cases, the outcomes of these MBPs are positive [3]. However, no specific correlations were offered between the adaptations and the outcomes of programs, which presents an opportunity for further research.

Regarding the content of adapted MBPs, creating and maintaining a robust container of safety is critical. The TBI staff and the mindfulness professionals acknowledged the importance of the container of safety. How this container of safety is constructed within the context of an MBP needs to be explored. This could include, for example, agreements around confidentiality, mutual respect and timing, providing meals, and practices that signify group commencement and closure. A realist review of the literature [3] found no references regarding the process of establishing a container of safety within MBPs, suggesting an avenue for future research.

The safety and containment of a group are interwoven with several other elements of adaptation. These might include ensuring that the mindfulness facilitator or co-facilitator is drawn from the same community as the participants to foster a sense of resonance [3, 39, 40, 42, 43]. The innate qualities and training of the facilitator, particularly the degree to which they embody the attitudes of mindfulness, kindness, calmness, humility and a welcoming approach, are critical to the acceptability and success of the program [7]. Safety and belonging might also be enhanced if the program is offered in the participants’ mother tongue or at least if a translator is available [38, 43].

Facilitators should also be flexible and responsive to emerging needs in the group. Appropriate facilitator recruitment and training before starting an MBP should ensure that the facilitator has the skills to establish and hold a robust container of safety throughout the MBP and the capacity to adapt to situations as they arise. Crane et al. [7] and Dutton et al. [39] highlight the relevance of mindfulness teacher training and offer guidelines, particularly concerning program adaptation.

Similar to structure, MBP content should also be relevant to the participants’ lives. A needs assessment should be undertaken among future participants and other stakeholders. Aligning the program content with the participant’s needs will increase attendance and retention and benefit participants.

Regarding the adaptation of mindfulness practices, most professionals reported that they used standard MBSR practices in their groups, and that the participants found these practices supportive. However, they suggested that the practices be framed and delivered in ways that feel less foreign and intimidating within communities unfamiliar with mindfulness. For example, “awareness of breath” might be referred to as “just noticing how you are breathing,” and yoga might be referred to as “stretching.” When adapting an MBP for TBI, it is unnecessary to omit any practices or introduce new ones, although facilitators should be sensitive to how the practice is presented and contextualized; for example, mindful movement could include traditional dancing.

Low resource contexts in South Africa are characterized by trauma at many levels, from personal traumatic events to the continuous daily trauma associated with survival amid poverty. Mindfulness professionals noted the importance of the facilitator being aware of the trauma experienced by their group participants and being sensitive to how the practices are offered and received. While none of the mindfulness professionals suggested specific trauma-sensitive practices, they noted that the practices should be introduced gradually and that the practices should be shorter than the standard MBSR practices so as not to overwhelm participants. MBPs should also be delivered with deep kindness, slowly building the capacity of participants for longer and more demanding practices.

While the literature does consider the value of a trauma-sensitive approach to mindfulness [3]^42^– [40, 44] further research is needed on the best approach for delivering MBPs within low-resource, trauma-dense contexts, particularly concerning the types and lengths of practices offered.

Facilitator training is important for MBPs in any context [7] and further research in this field would be valuable. Facilitators should be trained to embody the attitudes and qualities mindfulness, and to work in low-resource contexts. In addition, facilitators in low socioeconomic contexts, especially trauma-dense contexts, should be skilled in holding the container of safety, being flexible, and being trauma-sensitive.

Facilitators should also, if possible, have a cultural affinity with group participants, particularly the ability to resonate with the daily lived experiences of participants [40, 42, 43]. Additionally, facilitators should be committed to their practice – individually and in community – and be under regular supervision [45–47].

When working within low-resource contexts, the communication methods between the MBP facilitators and the participants should be considered. While participants from some communities may have access to smartphones and affordable or free WiFi, this was not the case among TBI vendors. Most of the TBI vendors in our study did not have access to smartphones or free WiFi. Using the existing communication structures between TBI and vendors may be most feasible in this setting. Regarding course materials, the TBI vendors appeared to have diverse literacy abilities, language use (spoken, read and written), and facilities to download audio files or print written material. Unless there is a substantial budget to fund translations, provide data and airtime, develop accessible written materials (potentially pictorial), and print notes and booklets, the most feasible approach with low resource settings seems to simply be to teach practices that can be practiced independently.

The participants had various concerns regarding the delivery language and the importance of translation within groups in low-resource contexts, particularly those as diverse as the TBI community. Some felt that translation was essential, whereas others felt that with relatively straightforward language use, a skilled facilitator could sufficiently communicate the essence of mindfulness.

### Limitations

The context-specific nature of this study may be seen as a limitation. While the findings may be transferable to MBPs within similar low-resource contexts both in South Africa and globally, they are not generalizable. However, as a qualitative study, this research is not intended to be generalizable.

The small sample size is a potential limitation to this study. A larger sample size including a greater number of vendors would have been preferable. However, only four vendors volunteered to be interviewed, of which one was interviewed independently and three as a focus group, to suit them all circumstantially. TBI is a small organization, and all four members of staff who work directly with the vendors on a daily basis were interviewed – two independently, and two as a focus group. While qualitative research does not require large sample sizes, the small sample size of this study might not reflect the broader range of opinions and experiences that a larger sample size might have offered.

A further limitation is the subjective nature of the stakeholders’ responses, which are based on their own experience. In addition, the four vendor participants had no knowledge or experience of mindfulness. They could therefore only comment on what would make them more or less likely to attend a program that would be supportive to them, rather than on a perceived benefit of mindfulness training.

It is also possible that the research outcomes may be influenced by the researcher’s biases and assumptions, reflected in the choice of interview questions and focus group questions, and by her interpretation of the data.

### Future directions

The adaptation of MBPs in low socioeconomic contexts is a developing field, and there are numerous opportunities for further research. These include strategies for creating a robust container of safety in low-resource contexts, particularly deepening participant engagement. Another focus is facilitator training, aimed at developing the skills to create a safe container in a low-resource, trauma-dense context while delivering MBPs that remain true to mindfulness as a well-being practice. Additionally, research can explore how to adapt practices, such as their type and length, to be most effective in low-resource, trauma-dense settings. Other areas include communication strategies for program delivery, translation, and venues encouraging attendance.

## Conclusion

The study explored the elements of an MBP that should be adapted for an MBP to be accessible, acceptable, and feasible to participants in a low-resource context. In particular, the study investigated adapting an MBP for the benefit of vendors of TBI print magazine in Cape Town, South Africa. The study participants were interviewed to gather their knowledge and experience of the various ways in which MBPs could be adapted to be applicable and beneficial to individuals within low-resource settings. The participants’ suggestions for adaptation offer an approach that honors the nonnegotiable elements that define an MBP and addresses the negotiable elements that might be adapted to meet the specific needs and requirements of the vendor community. Further implementation and evaluation research should be undertaken to assess whether an adapted program may be of benefit to the vendors.

## Supplementary Information

Supplementary Material 1.

## Data Availability

No datasets were generated or analysed during the current study.

## Abbreviations

HR: Human Resources
IMISA: Institute for Mindfulness South Africa
MBSR: Mindfulness-based stress reduction
MBCT: Mindfulness-based cognitive therapy
MBP: Mindfulness-Based Programmes
MBTR-R: Mindfulness-based trauma recovery for refugees
TBI: The Big Issue
MP: Mindfulness professional
NPO: Nonprofit organization
TRE: Tension and Trauma Releasing Exercises
