# Supplementary material for: Considerations for sociocultural adaptations of a mindfulness-based program within a low socioeconomic setting in Cape Town, South Africa
Source: BMC Complement Med Ther. 2025 Dec 30;25:453. doi: 10.1186/s12906-025-05122-3 (PMC12754989; doi:10.1186/s12906-025-05122-3)
Supplement: Supplementary file 1 — Supplementary Material 1. [file 12906_2025_5122_MOESM1_ESM.docx]

## INTERVIEW QUESTIONS

INTERVIEW QUESTIONS FOR “MINDFULNESS PROFESSIONAL” STAKEHOLDERS

(to be administered verbally during interviews)

- After preliminary greetings, ground the interview with a short mindfulness sitting practice.

Allowing the body to settle in whatever position it is in right now, and closing the eyes if that feels right for you. Feeling the sense of the feet on the floor, the sit bones on the chair if you are sitting, the sense of the spine rising upwards, arms hanging softly as the sides. Taking a deep breath in, and then a long slow breath out, pausing to allow whatever else has happened thus far in the day to settle. Another deep breath in, and another slow breath out. Noticing the sense of the heart beating in the body. Feeling the flow of the breath in and of the breath out. And, whenever your feel ready, allowing the eyes to float open again. Welcome!

- Introductory question:

*Please could you share briefly who you are, where you are based and what your work is.*

- One ‘core’ question for “mindfulness professional” stakeholders:

*From your experience of working with mindfulness within low socio-economic settings, what have you learned about adapting these initiatives to be of greater benefit to the participants?*

Closing move:

- *In writing up the research, we will be using pseudonyms or codes to ensure participant confidentiality.*

*We would like to offer our heartfelt gratitude to you for giving up your time for this interview; for being willing to share your work in the field; and for contributing in this way to what will potentially make the benefits of mindfulness practice available in settings where daily life is a struggle and where resources to help mitigate this struggle are limited. This is greatly appreciated - thank you very much.*

INTERVIEW QUESTIONS FOR “TBI STAFF” STAKEHOLDERS

(to be administered verbally during interviews)

- As this research is grounded in mindfulness, would it be okay if we start with a very brief practice, before we get going with the interview?

Allowing the body to settle in whatever position it is in right now, and closing the eyes if that feels right for you. Feeling the sense of the feet on the floor, the sit bones on the chair if you are sitting, the sense of the spine rising upwards, arms hanging softly as the sides. Taking a deep breath in, and then a long slow breath out, pausing to allow whatever else has happened thus far in the day to settle. Another deep breath in, and another slow breath out. Noticing the sense of the heart beating in the body. Feeling the flow of the breath in and of the breath out. And, whenever your feel ready, allowing the eyes to float open again, present to this shared time together. Welcome!

- Introductory question for “TBI management” stakeholders:

*Please could you share briefly who you are, what your work is.*

- One ‘core’ question for “TBI management” stakeholders:

*From your experience of working with the TBI community, please could you share your thoughts on how an MBP oriented towards enhancing well-being could be tailored to be of most benefit to the participants?*

*What factors should be considered when adapting and implementing a programme in this community?*

- Closing move:

*In writing up the research, we will be using pseudonyms or codes to ensure participant confidentiality.*

*We would like to offer our heartfelt gratitude to you for giving up your time for this interview; for being willing to share your work in the field; and for contributing in this way to what will potentially make the benefits of mindfulness practice available in settings where daily life is a struggle and where resources to help mitigate this struggle are limited. This is greatly appreciated - thank you very much.*

INTERVIEW QUESTIONS FOR “TBI VENDOR” STAKEHOLDERS

(to be administered verbally during interviews)

Background questions:

- What is your name?
- What is your work?
- Where do you live and what is your neighbourhood / community like?
- How far is your home from where you sell TBI and how do you travel between home and work?
- What do you struggle with in your day-to-day life?

Core questions:

- *What would make it more likely for you to choose to join a program?*
- *What would make it difficult for you to attend a programme?*
- Closing move:

*In writing up the research, we will be using pseudonyms or codes to ensure participant confidentiality.*

*We would like to offer our heartfelt gratitude to you for giving up your time for this interview and for contributing to this study. Your contribution towards potentially making the benefits of mindfulness practice available to all is greatly appreciated - thank you very much.*
